# The Distribution of Macrophages with a M1 or M2 Phenotype in Relation to Prognosis and the Molecular Characteristics of Colorectal Cancer

**DOI:** 10.1371/journal.pone.0047045

**Published:** 2012-10-15

**Authors:** Sofia Edin, Maria L. Wikberg, Anna M. Dahlin, Jörgen Rutegård, Åke Öberg, Per-Arne Oldenborg, Richard Palmqvist

**Affiliations:** 1 Department of Medical Biosciences, Pathology, Umeå University, Umeå, Sweden; 2 Department of Surgical and Perioperative Sciences, Surgery, Umeå University, Umeå, Sweden; 3 Department of Integrative Medical Biology, Histology and Cell Biology, Umeå University, Umeå, Sweden; City of Hope National Medical Center and Beckman Research Institute, United States of America

## Abstract

High macrophage infiltration has been correlated to improved survival in colorectal cancer (CRC). Tumor associated macrophages (TAMs) play complex roles in tumorigenesis since they are believed to hold both tumor preventing (M1 macrophages) and tumor promoting (M2 macrophages) activities. Here we have applied an immunohistochemical approach to determine the degree of infiltrating macrophages with a M1 or M2 phenotype in clinical specimens of CRC in relation to prognosis, both in CRC in general but also in subgroups of CRC defined by microsatellite instability (MSI) screening status and the CpG island methylator phenotype (CIMP). A total of 485 consecutive CRC specimens were stained for nitric oxide synthase 2 (NOS2) (also denoted iNOS) as a marker for the M1 macrophage phenotype and the scavenger receptor CD163 as a marker for the M2 macrophage phenotype. The average infiltration of NOS2 and CD163 expressing macrophages along the invasive tumor front was semi-quantitatively evaluated using a four-graded scale. Two subtypes of macrophages, displaying M1 (NOS2^+^) or M2 (CD163^+^) phenotypes, were recognized. We observed a significant correlation between the amount of NOS2^+^ and CD163^+^ cells (*P*<0.0001). A strong inverse correlation to tumor stage was found for both NOS2 (*P*<0.0001) and CD163 (*P*<0.0001) infiltration. Furthermore, patients harbouring tumors highly infiltrated by NOS2^+^ cells had a significantly better prognosis than those infiltrated by few NOS2^+^ cells, and this was found to be independent of MSI screening status and CIMP status. No significant difference was found on cancer-specific survival in groups of CRC with different NOS2/CD163 ratios. In conclusion, an increased infiltration of macrophages with a M1 phenotype at the tumor front is accompanied by a concomitant increase in macrophages with a M2 phenotype, and in a stage dependent manner correlated to a better prognosis in patients with CRC.

## Introduction

Inflammatory cells are present in the tumor microenvironment of most cancers and have been reported to affect the milieu of inflammatory mediators and cell proliferation signals, angiogenesis and tissue remodelling in ways that could promote tumor progression [1]–[3]. Tumor-associated macrophages (TAMs) and their roles in tumor invasion and metastasis have been discussed in several recent reviews [4]–[8]. In general, TAMs are found within and surrounding most tumors and can, when activated, release numerous factors that could influence the behaviour of tumor cells and other cells of tumor stroma. The ability of macrophages to adapt to their environment has lead to the identification of two main polarized phenotypes of macrophages [7], [9]. In brief, the classically activated M1 macrophages are characterized by the expression of nitric oxide synthase 2 (NOS2) (also denoted iNOS), as well as many pro-inflammatory cytokines (e.g IL1β, IL6, IL12, IL23 and TNF) and are reported to have a high bactericidal and tumoricidal capacity. The main functions of the alternatively activated M2 macrophages are instead to scavenge debris and promote tissue repair, but they also have immune regulatory functions. Many of the factors produced by M2 macrophages act in favour of tumor progression, stimulating tumor growth, (e.g. epidermal growth factor (EGF), fibroblast growth factor 1 (FGF1) and transforming growth factor beta 1 (TGFβ1), angiogenesis (e.g. vascular endothelial growth factor A (VEGFA)) and matrix remodelling (e.g. FGF1, fibrin and matrix metallopeptidases (MMPs)). In addition, M2 macrophages also produce immune regulatory factors (e.g. IL10 and TGFβ1) that dampen the immune response.

TAMs are often found to have a M2 phenotype and have been associated with a decreased survival in patients with e.g. melanoma [10], [11], breast [12], [13], kidney [14] and bladder cancer [15], [16]. However, this is not true for all cancers. We and others have previously shown that an increased density of macrophages in CRC is correlated to a better prognosis [17]–[20]. Also stomach cancer patients have been reported to have a better prognosis with a high number of TAMs [21], [22]. The results on prostate [23], [24], lung [25]–[29] and endometrial cancer [30]–[32] are however conflicting. It is becoming increasingly evident that macrophages can play different roles in tumorigenesis dependent on tissue and cancer type. It is interesting to speculate that the different roles played by macrophages in various cancers could involve variations in the balance between M1 and M2 phenotypes (tumor prevention vs. tumor promotion), driven by factors in the tumor microenvironment of individual cancers. There could also be variations within certain cancer types.

CRCs have been subtyped according to their microsatellite (MSI) status and the CpG island methylator phenotype (CIMP). Approximately 15% of CRCs are defined as microsatellite unstable (MSI), a phenotype caused by defects in DNA mismatch repair, which is in contrast to microsatellite stable (MSS) CRCs [33]. In sporadic CRCs, MSI has been highly associated with CIMP [34]. CIMP can be classified as CIMP-high or CIMP-low according to the hypermetylation status of CpG islands in a set of genes that are unmethylated in normal colorectal tissue or in CIMP-negative tumors [35]–[38].

In the present study, the distribution of different subtypes of macrophages was evalutated in 485 clinical specimens of CRC, using nitric oxide synthase 2 (NOS2) as a marker for the M1 macrophage phenotype and the scavenger receptor CD163 as a marker for the M2 macrophage phenotype. The infiltration of NOS2^+^ and CD163^+^ cells was related to clinicopathologic and molecular variables, as well as prognosis, both in the complete CRC cohort and in subgroups of CRC defined by MSI screening status and CIMP status. We could conclude that infiltration of M1 macrophages in CRC is accompanied by infiltrating M2 macrophages and correlated to improved survival in a stage dependent manner in CRC, and that this is independent of MSI screening status and CIMP status.

## Materials and Methods

### Ethics Statement

The handling of tissue samples and patient data in the present study was approved by the research ethical committee at Umeå University Hospital (Regional Ethical Review Board in Umeå, Sweden), including the procedure whereby patients verbally gave their informed consent. This consent was documented in each patient record, and considered by the Ethics Committee to be sufficient. Tissue samples were registered as a case number and year in a database used for the analyses, with no names or personal identification number indicated.

### Study population

Clinical specimens from patients of the Colorectal Cancer in Umeå Study (CRUMS) [39], surgically resected for CRC were collected between 1995 and 2003 at the department of Surgery, Umeå University Hospital, Umeå, Sweden. From all patients, formalin-fixed paraffin embedded tissue was sampled and pathological variables were characterized by one pathologist by reviewing routinely stained sections. Clinical data, including survival data, were obtained by one surgeon by reviewing the patient records. A total of 485 patients (300 colon cancers, 180 rectal cancers, and 5 not specified subsite within the colorectum) were included in the study. With 28 patients missing information on either NOS2 or CD163 expression, 474 specimens for NOS2 and 468 specimens for CD163 were available for analysis. Adjuvant chemotherapy was administered to 68 (14.0%) patients. Preoperative radiation therapy was administered to 108 (60.0%) rectal cancer patients of whom 83 received 5×5 Gy, and 25 received 25×2 Gy. For survival analyses, 37 patients were excluded due to incomplete follow-up data or due to death by perioperative complications. For survival analyses scores regarding the NOS2/CD163 ratio, a total of 422 patients were available.

### Immunohistochemistry and immunoflourescense

Specimens were fixed in 4% formaldehyde and embedded in paraffin, according to routine procedures at the department of Clinical Pathology, Umeå University Hospital, Umeå Sweden. One 4-µm section from each patient was cut, dried, de-waxed and rehydrated. Slides were then subjected to heat-mediated antigen retrieval using Diva solution (Biocare Medical, Concord, CA) in a Decloaker™ pressure cooker. For immunohistochemical procedures, a semiautomatic staining machine (Ventana ES, Ventana Inc., Tuscon, AZ) was used. Anti-CD163 monoclonal antibody (Novacastra) was used at a dilution of 1∶100, and anti-NOS2 polyclonal antibody (Abcam) was used at a dilution of 1∶50. The slides were counterstained with hematoxylin.

For evaluation, slides were reviewed under light microscope. Immunohistochemical staining was evaluated as most representative area at the invasive front and assessed as no/weak (score 1), moderate (score 2), strong/robust (score 3) and massive infiltration (score 4) according to Forssell et al. [18]. The specimens were evaluated two times by the same observer, and discordant cases were reviewed a third time, followed by a conclusive judgement. For immunoflourescense, anti-CD163 monoclonal antibody, anti-NOS2 rabbit polyclonal antibody and anti-CD68 rabbit polyclonal antibody (GeneTex Inc) was used at a dilution of 1∶50, and anti-CD68 mouse monoclonal antibody (Dako) was used at a dilution of 1∶400. For block, the tissue sections were treated with PBS containing 10% normal goat serum and 0.4% Triton X-100 for 20 minutes, followed by wash in wash buffer (PBS; 0.2% Triton X-100; 0.2% bovine serum albumin). Next, the slides were incubated with primary antibody (in PBS; 0.1% Triton X-100) for 1 hour at room temperature, after which they were washed in wash buffer. The slides were further incubated with DAPI at a dilution of 1∶1000 and secondary anti-rabbit IgG Alexa®488 and anti-rabbit IgG Alexa®555 antibodies (Invitrogen) diluted 1∶400 (in PBS; 0.1% Triton X-100) for 1 hour. After additional washes, the slides were mounted in Vectashield mounting medium (Vector Laboratories) and viewed using a Nikon D-Eclipse C1 confocal microscope with oil immersion and a 40× objective.

### MSI screening status and CIMP status

MSI screening status was determined by immunohistochemistry as previously described [39]. A positive MSI screening status (MSI) was assigned to tissue samples with tumor cells lacking nuclear staining for one or more of the proteins MLH1, MSH2, MSH6, or PMS2, this in contrast to a negative screening status (MSS), where positive tumor nuclei were present expressing all four markers. CIMP status was determined according to hypermethylation of an eight-gene panel (*CDKN2A*, *MLH1*, *CACNA1G*, *NEUROG1*, *RUNX3*, *SOCS1*, *IGF2*, *and CRABP1*) by the MethyLight method (quantitative real-time PCR) with previously described primer and probe sequences [39], [40]. The following number of hypermethylated genes defined CIMP-negative tumors, 0 genes; CIMP-low tumors, 1–5 genes; and CIMP high tumors, 6–8 genes.

### Statistical analyses

Statistical analyses were performed using PASW Statistics 18 (SPSS Inc., Chicago, Illinois, USA). Cross-tabulations were analyzed with Fischer's exact test and linear relationships with the exact linear-by-linear association test. Kaplan-Meier survival analysis was used to estimate cancer-specific survival, and comparisons between groups were performed with the log-rank test. Cancer-specific survival was defined as death with known disseminated or recurrent disease. Multivariate survival analyses were performed by using Cox proportional hazard models. *P*<0.05 was considered statistically significant.

## Results

### Expression of M1 and M2 macrophage markers

**Figure 1:**
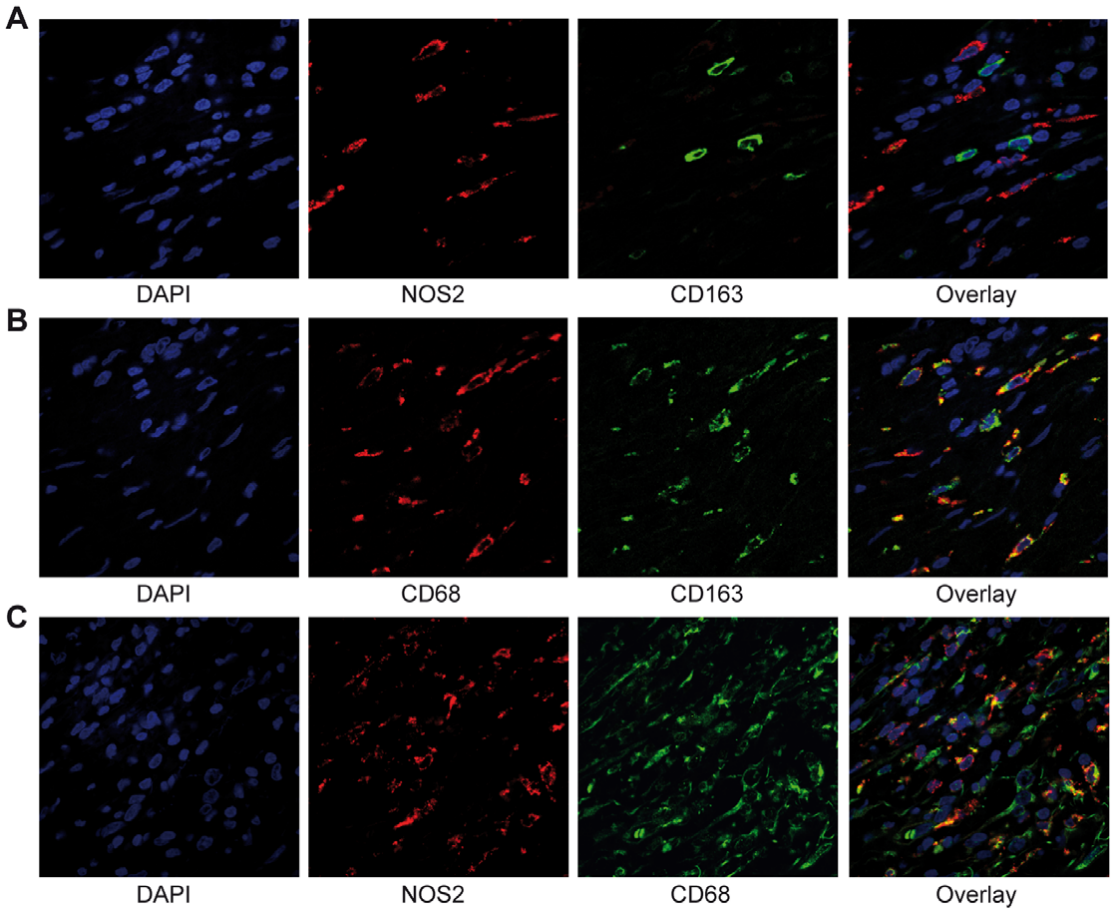
NOS2 and CD163 distinguish between different macrophage phenotypes. Confocal images of immunoflourescent stainings in CRC of (A) NOS2 (red) and CD163 (green), (B) CD68 (red) and CD163 (green) and (C) NOS2 (red) and CD68 (green). Nuclei are revealed by DAPI staining (blue), and overlay is flourescense collected by all channels.

NOS2 was selected as a marker for macrophages with a M1 phenotype and CD163 as a marker for macrophages with a M2 phenotype. To validate whether NOS2 and CD163 were markers, able to separate between distinct populations of macrophages, specimens of 10 CRC patients were randomly selected and the distribution of NOS2 and CD163 was analyzed by double immunoflourescent staining followed by confocal microscopy. NOS2 and CD163 was found to be primarily expressed by different populations of macrophages (Figure 1A). A small over-lap could however be identified, which is in line with the plastic nature of macrophages. Macrophages that highly expressed one of the markers, however, consistently did not express the second marker. This verifies NOS2 and CD163 as markers that can be used to distinguish between different subpopulations of macrophages displaying mainly M1 or M2 phenotypes, respectively. Furthermore, NOS2 and CD163 expression was found in cells that also expressed the macrophage marker CD68 (Figure 1B and C).

**Figure 2:**
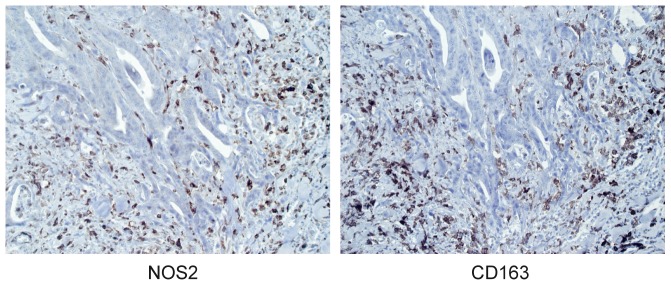
NOS2 and CD163 immunoreactivity in patient samples. Representative light microscopic images of immunohistochemical stainings of NOS2 and CD163 in consecutive sections of the same CRC tumor sample.

Expression of NOS2 and CD163 was semi-quantitatively evaluated in specimens from 485 CRC patients using immunohistochemistry according to a previously documented four-graded scale [18]. Representative stainings of infiltrating NOS2^+^ or CD163^+^ macrophages are shown i figure 2. Approximately 70% of all tumors displayed a modest to massive infiltration of NOS2^+^ and CD163^+^ cells (score 2–4), while the remaining showed weak or no infiltration (score 1). The majority of NOS2 and CD163 expression was found in cells located in the tumor stroma, with the highest density along the invasive tumor front.

### The relationship between NOS2^+^ and CD163^+^ macrophages

The frequencies of infiltrating NOS2^+^ and CD163^+^ macrophages at the tumor front are presented in table 1. Infiltrating macrophages expressing NOS2 or CD163 were highly positively correlated (*P*<0.0001). The amounts of CD163^+^ cells at the tumor front, however, were frequently higher than that of NOS2^+^ cells.

**Table 1:** Cross-tabulation between NOS2 and CD163.

	NOS2				*P*
CD163	1	2	3	4	
**1**	79 (59.4)	51 (38.3)	3 (2.3)	0 (0.0)	<0.0001*
**2**	64 (32.5)	105 (53.3)	28 (14.2)	0 (0.0)	
**3**	10 (8.8)	52 (46.0)	47 (41.6)	4 (3.5)	
**4**	0 (0.0)	4 (28.6)	7 (50.0)	3 (21.4)	

* Exact linear-by-linear association test.

### Association between infiltrating NOS2^+^ and CD163^+^ macrophages and clinicopathologic and molecular parameters

The scores of infiltrating NOS2^+^ and CD163^+^ macrophages in CRC specimens were correlated to various clinicopathologic variables (Table 2). When including all tumors, no relation of NOS2^+^ or CD163^+^ macrophage infiltration was found to gender, age, grade, growth pattern, adjuvant chemotherapy or preoperative radiotherapy. A weak linear trend was found for increased infiltration of NOS2^+^ macrophages from the ceacum to the rectum (*P* = 0.043). While no significant association of NOS2 expression was found with tumor type, a significant association with tumor type was found for CD163 expression (*P* = 0.005), with CD163 infiltrated tumors more often having a non-mucinous histology. A strong inverse association with tumor stage was found for both NOS2^+^ (*P*<0.0001) and CD163^+^ (*P*<0.0001) macrophage infiltration.

**Table 2:** NOS2 and CD163 expression at the tumor invasive front in relation to clinicopathologic characteristics in CRC.

	NOS2				*P*	CD163				*P*
	1	2	3	4		1	2	3	4	
**Frequency (%)**	158 (33.3)	221 (46.6)	86 (18.1)	9 (1.9)		138 (29.5)	202 (43.2)	114 (24.4)	14 (3.0)	
**Gender, n (%)**					0.543					0.479
Male	80 (30.5)	128 (48.9)	49 (18.7)	5 (1.9)		71 (26.9)	115 (43.6)	69 (26.1)	9 (3.4)	
Female	78 (36.8)	93 (43.9)	37 (17.5)	4 (1.9)		67 (32.8)	87 (42.6)	45 (22.1)	5 (2.5)	
**Age, n (%)**					0.087/0.378*					0.106/0.651*
≤59	24 (26.1)	45 (48.9)	21 (22.8)	2 (2.2)		28 (29.5)	32 (33.7)	31 (32.6)	4 (4.2)	
60–69	41 (34.7)	51 (43.2)	24 (20.3)	2 (1.7)		33 (28.2)	55 (47.0)	27 (23.1)	2 (1.7)	
70–79	69 (42.1)	69 (42.1)	22 (13.4)	4 (2.4)		57 (35.4)	68 (42.2)	30 (18.6)	6 (3.7)	
≥80	24 (24.0)	56 (56.0)	19 (19.0)	1 (1.0)		20 (21.1)	47 (49.5)	26 (27.4)	2 (2.1)	
**Localization, n (%)**					0.824/0.043*					0.919/0.279*
Caecum	16 (32.7)	27 (55.1)	6 (12.2)	0 (0.0)		17 (34.0)	22 (44.0)	10 (20.0)	1 (2.0)	
Ascending colon	34 (44.2)	28 (36,4)	14 (18.2)	1 (1.3)		28 (35.9)	29 (37.2)	18 (23.1)	3 (3.8)	
Transverse colon	7 (33.3)	12 (57.1)	2 (9.5)	0 (0.0)		4 (19.0)	10 (47.6)	7 (33.3)	0 (0.0)	
Splenic flexure	5 (33.3)	7 (46.7)	3 (20.0)	0 (0.0)		3 (21.4)	8 (57.1)	3 (21.4)	0 (0.0)	
Descending colon	3 (23.1)	7 (53.8)	3 (23.1)	0 (0.0)		2 (15.4)	8 (61.5)	3 (23.1)	0 (0.0)	
Sigmoid colon	41 (34.5)	53 (44.5)	22 (18.5)	3 (2.5)		38 (32.5)	49 (41.9)	27 (23.1)	3 (2.6)	
Rectum	52 (29.5)	85 (48.3)	34 (19.3)	5 (2.8)		46 (27.1)	74 (43.5)	43 (25.3)	7 (4.1)	
**Stage, n (%)**					0.002<0.0001*					<0.0001/<0.0001*
I	17 (23.6)	33 (45.8)	21 (29.2)	1 (1.4)		10 (14.3)	32 (45.7)	23 (32.9)	5 (7.1)	
II	53 (28.3)	88 (47.1)	41 (21.9)	5 (2.7)		48 (26.5)	70 (38.7)	58 (32.0)	5 (2.8)	
III	35 (36.5)	48 (50.0)	13 (13.5)	0 (0.0)		26 (26.8)	52 (53.6)	17 (17.5)	2 (2.1)	
IV	51 (46.8)	47 (43.1)	10 (9.2)	1 (0.9)		52 (46.8)	43 (38.7)	14 (12.6)	2 (1.8)	
**Grade, n (%)**					0.301					0.227
Low	66 (28.8)	114 (49.8)	45 (19.7)	4 (1.7)		61 (26.6)	100 (43.7)	63 (27.5)	5 (2.2)	
High	87 (36.7)	106 (44.7)	39 (16.5)	5 (2.1)		76 (32.9)	99 (42.9)	48 (20.8)	8 (3.5)	
**Growth pattern, n (%)**					0.183					0.663
Pushing	60 (38.7)	70 (45.2)	24 (15.5)	1 (0.6)		47 (30.5)	70 (45.5)	34 (22.1)	3 (1.9)	
Infiltrating	94 (30.4)	147 (47.6)	60 (19.4)	8 (2.6)		90 (29.5)	127 (41.6)	77 (25.2)	11 (3.6)	
**Histology type, n (%)**					0.348					0.005
Non-mucinous	127 (32.0)	188 (47.4)	73 (18.4)	9 (2.3)		105 (27.0)	169 (43.4)	103 (26.5)	12 (3.1)	
Mucinous	29 (41.4)	31 (44.3)	10 (14.3)	0 (0.0)		32 (44.4)	30 (41.7)	8 (11.1)	2 (2.8)	
**Adjuvant chemotherapy, n (%)**					0.245					0.814
No	127 (31.8)	192 (48.1)	72 (18.0)	8 (2.0)		119 (30.3)	168 (42.7)	95 (24.2)	11 (2.8)	
Yes	28 (43.1)	25 (38.5)	12 (18.5)	0 (0.0)		17 (26.6)	27 (42.2)	17 (26.6)	3 (4.7)	
**Preoperative radiation therapy** † **, n (%)**					0.326					0.941
No	23 (32.4)	34 (47.9)	14 (19.7)	0 (0.0)		18 (26.5)	31 (45.6)	17 (25.0)	2 (2.9)	
Yes	29 (27.6)	51 (48.6)	20 (19.0)	5 (4.8)		28 (27.5)	43 (42.2)	26 (25.5)	5 (4.9)	

The following number of missing cases were present in analyses for NOS2 and CD163, respectively: localization, 4 and 5; stage, 10 and 9; grade, 8 and 8; growth pattern, 10 and 9; histology type, 7 and 7; adjuvant chemotherapy, 10 and 11, and preoperative radiation therapy; 4 and 10. Unless otherwise indicated, Fisher's exact test was used for categorical variables.

* Exact linear-by-linear association test was used to test for linear relationship between variables.

† Preoperative radiation therapy in rectal cancers only.

When relating infiltrating NOS2^+^ or CD163^+^ macrophages to molecular parameters (Table 3), no correlation of NOS2^+^ or CD163^+^ macrophage infiltration was found to either MSI screening status or CIMP status. When combining MSI screening status with CIMP status however, CD163^+^ macrophage infiltration was found to be significantly lower in CIMP-high tumors compared with CIMP-negative or CIMP-low tumors among the group of MSS tumors (*P* = 0.042).

**Table 3:** NOS2 and CD163 expression at the tumor invasive front in relation to molecular characteristics in CRC.

		NOS2				*P*	CD163				*P*
		1	2	3	4		1	2	3	4	
**MSI screening status** † **, n (%)**						0.439					0.182
MSI		19 (26.4)	36 (50.0)	16 (22.2)	1 (1.4)		17 (23.6)	29 (40.3)	22 (30.6)	4 (5.6)	
MSS		137 (35.2)	178 (45.8)	67 (17.2)	7 (1.8)		117 (30.7)	166 (43.6)	89 (23.4)	9 (2.4)	
**CIMP status** § **, n (%)**						0.297					0.115
CIMP-negative		71 (29.8)	120 (50.4)	40 (16.8)	7 (2.9)		65 (28.0)	104 (44.8)	52 (22.4)	11 (4.7)	
CIMP-low		66 (37.7)	73 (41.7)	35 (20.0)	1 (0.6)		58 (33.0)	69 (39.2)	48 (27.3)	1 (0.6)	
CIMP-high		19 (32.2)	28 (47.5)	11 (18.6)	1 (1.7)		14 (24.1)	28 (48.3)	14 (24.1)	2 (3.4)	
**Combined MSI screening and CIMP status, n (%)**						0.735					0.705
MSI CIMP-negative		2 (13.3)	10 (66.7)	3 (20.0)	0 (0.0)		5 (33.3)	6 (40.0)	3 (20.0)	1 (6.7)	
MSI CIMP-low		6 (33.3)	9 (50.0)	3 (16.7)	0 (0.0)		5 (27.8)	5 (27.8)	7 (38.9)	1 (5.6)	
MSI CIMP-high		11 (28.2)	17 (43.6)	10 (25.6)	1 (2.6)		7 (17.9)	18 (46.2)	12 (30.8)	2 (5.1)	
						0.164					0.042
MSS CIMP-negative		68 (31.1)	109 (49.8)	36 (16.4)	6 (2.7)		60 (28.2)	96 (45.1)	48 (22.5)	9 (4.2)	
MSS CIMP-low		60 (40.0)	59 (39.3)	30 (20.0)	1 (0.7)		50 (33.3)	60 (40.0)	40 (26.7)	0 (0.0)	
MSS CIMP-high		8 (42.1)	10 (52.6)	1 (5.3)	0 (0.0)		6 (35.3)	10 (58.8)	1 (5.9)	0 (0.0)	

The following number of missing cases were present in analyses for NOS2 and CD163, respectively: MSI screening status, 13 and 15; CIMP status, 2 and 2, and combined MSI screening and CIMP status, 14 and 16. Fisher's exact test was used for categorical variables.

† Cases lacking nuclear staining of tumor cells for at least one of MLH1, MSH2, MSH6, or PMS2 were considered to have a positive MSI screening status.

§ Phenotype determined according to hypermethylation of an eight-gene panel with the follwing number of hypermethylated genes found for CIMP-negative, 0 genes; CIMP-low, 1–5 genes, and CIMP-high, 6–8 genes. MSI, microsatellite instability; MSS, microsatellite stable; CIMP, CpG island methylator phenotype.

### Prognostic importance of infiltrating NOS2^+^ and CD163^+^ macrophages

**Figure 3:**
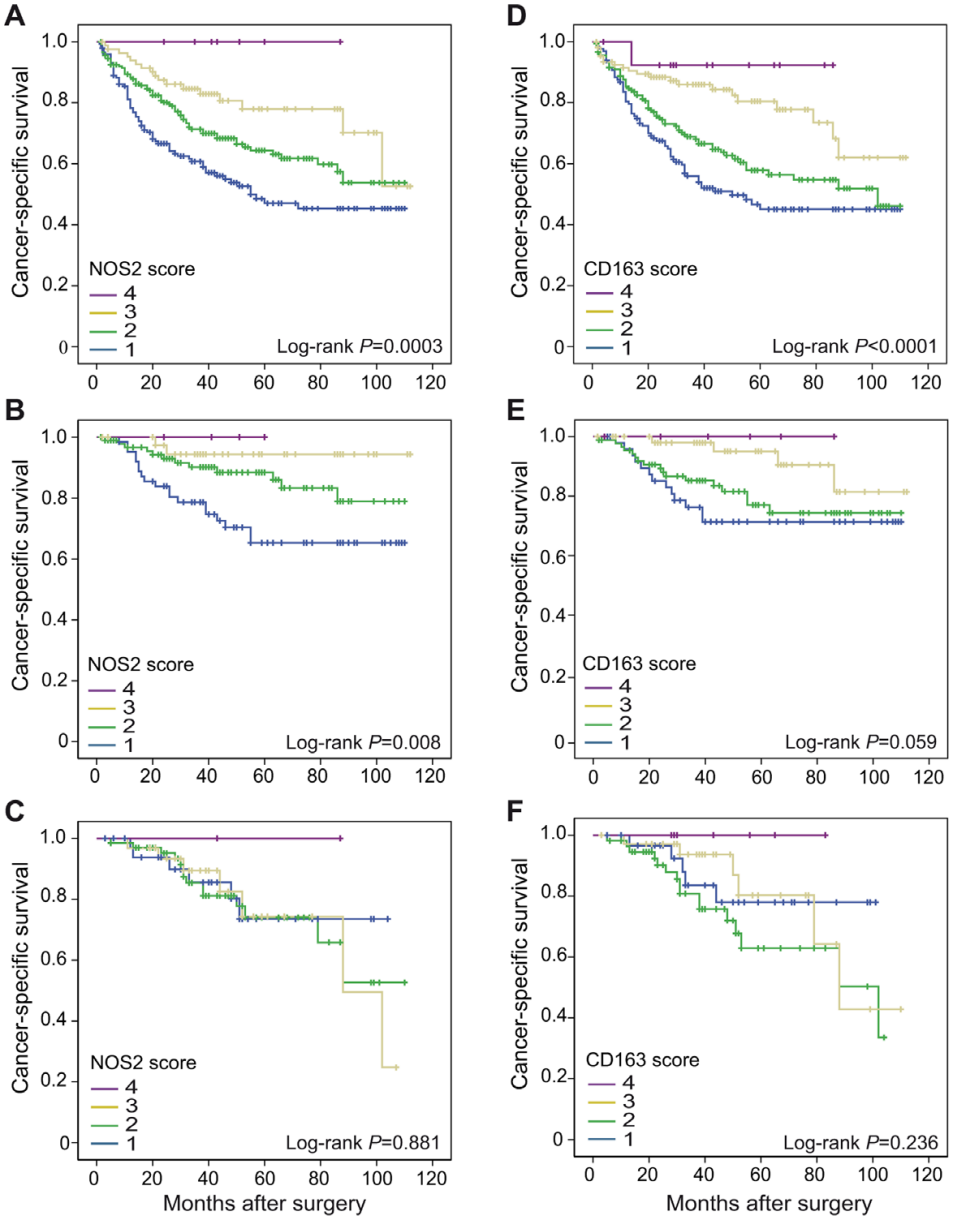
Cancer-specific survival in CRC patients. CRC cases were scored for NOS2 (A–C) and CD163 (D–F) expression, score 1–4. Shown are Kaplan-Meier plots of cancer-specific survival in (A and D) all CRCs, (B and E) potentially curatively resected colon cancers, and (C and F) potentially curatively resected rectal cancers. Log-rank tests were used to calculate *P* values.

To assess the prognostic impact of macrophage infiltration, we compared overall cancer-specific survival in patients with different scores of infiltrating NOS2^+^ or CD163^+^ macrophages. Figure 3 shows Kaplan-Meier plots of cancer-specific survival in CRUMS patients with different levels of infiltrating NOS2^+^ and CD163^+^ macrophages. An increased infiltration of NOS2^+^ macrophages at the tumor front was highly significantly associated with an improved prognosis (Log-rank *P* = 0.0003) (Figure 3A). A similar association was seen also for CD163^+^ macrophages (Log-rank *P*<0.0001) (Figure 3D). In potentially curatively resected CRCs (i.e excluding patients with distant metastases or non-radical surgery) the significance of the association between NOS2^+^ macrophage infiltration and prognosis was lost (Log-rank *P* = 0.132). However, in this group the significance of NOS2^+^ macrophage infiltration and prognosis was restored when separating cases of colon cancer from rectal cancers, Log-rank *P* = 0.008 in colon compared to Log-rank *P* = 0.881 in rectum (Figure 3B and C). For the corresponding analysis of CD163^+^ macrophage infiltration in curatively resected CRCs a similar tendency was found (Log-rank *P* = 0.034 in all CRCs; Log-rank *P* = 0.059 in colon; Log-rank *P* = 0.236 in rectum) (Figure 3E and F).

Because of the strong correlation between tumor stage and the expression of NOS2 and CD163 we performed multivariate Cox proportional hazard models including the variables gender, age, localization, tumor stage, and one macrophage marker, respectively. Hazard ratios (HRs) for both NOS2 (HR 0.67, 95% CI 0.40–1.12, *P* = 0.12) and CD163 (HR 0.66, 95% CI 0.42–1.06, *P* = 0.087) indicated a protective effect but did not reach statistical significance, emphasizing the stage dependence.

**Figure 4:**
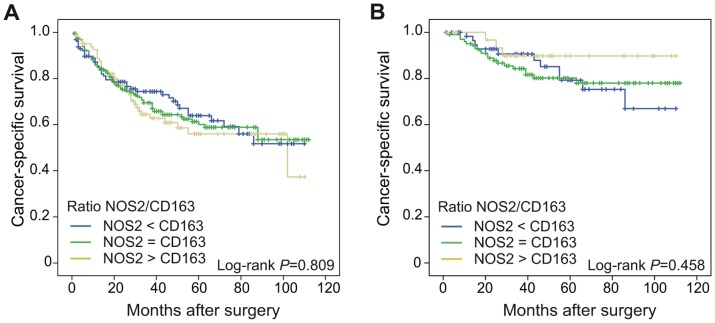
NOS2/CD163 ratios and cancer-specific survival in CRC. Cancer-specific survival of CRC cases scored for the NOS2/CD163 ratio. Shown are Kaplan-Meier plots of the NOS2/CD163 ratio in (A) all CRCs, or (B) potentially curatively resected colon cancers. Log-rank tests were used to calculate *P* values.

The possible effect of variations in NOS2/CD163 ratio on patient survival was also analyzed. No significant difference was seen on cancer-specific survival in CRC in relation to the NOS2/CD163 ratio, neither in all CRC cases (Figure 4A) nor in the selected group of potentially curatively resected colon cancers (Figure 4B). Furthermore, the NOS2/CD163 ratio was not significantly associated with survival in multivariate analysis (data not shown).

### Prognostic importance of infiltrating NOS2^+^ and CD163^+^ macrophages according to MSI screening status and CIMP status

To further analyze the prognostic value of macrophage infiltration, we compared overall cancer-specific survival within different subgroups of CRC defined by MSI screening status and CIMP status.

**Figure 5:**
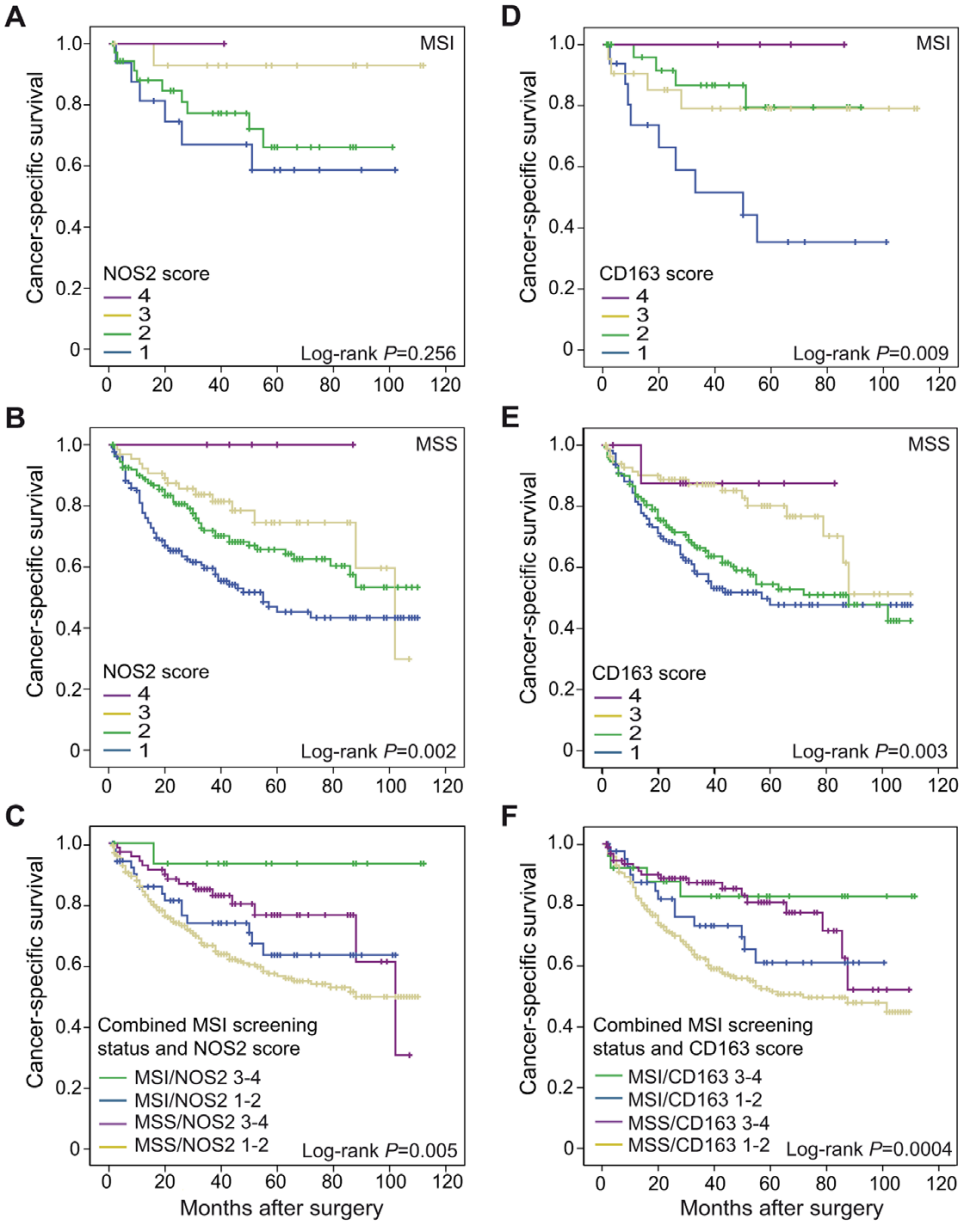
Cancer-specific survival in subgroups of CRC arranged according to MSI screening status. Subgroups of CRC were arranged according to MSI screening status and scored for NOS2 (A–C) and CD163 (D–F) expression, score 1–4. Shown are Kaplan-Meier plots of cancer-specific survival in (A and D) MSI cases, (B and E) MSS cases, and (C and F) combined MSI screening status and NOS2 or CD163, score 1–2 or 3–4, respectively. Log-rank tests were used to calculate *P* values.

MSI cases of CRC are found to have a slightly better prognosis compared to MSS cases [33]. Macrophage infiltration was found to be a prognostic factor in subgroups of both MSI (Figure 5A and D) and MSS cases (Figure 5B and E). The prognostic value of NOS2^+^ macrophage infiltration did not reach significance in MSI cases (Log-rank *P* = 0.256), but it did so in MSS cases (Log-rank *P* = 0.002). CD163^+^ macrophage infiltration was significant for prognosis in both MSI (Log-rank *P* = 0.009) and MSS (Log-rank *P* = 0.003) cases. When combining MSI screening status with low (score 1–2) or high (score 3–4) infiltration of NOS2^+^ or CD163^+^ macrophages, significant effects on prognosis was found for both NOS2^+^ (Log-rank *P* = 0.005) and CD163^+^ (Log-rank *P* = 0.0004) macrophage infiltration (Figure 5C and F). The most favourable prognosis was found in MSI cases highly infiltrated by macrophages, in particular by NOS2^+^ macrophages. MSS cases with low macrophage infiltration displayed the worst prognosis (Figure 5C and F). No significant differences on prognosis were found between MSI and MSS cases within subgroups with low or high infiltration of NOS2^+^ or CD163^+^ macrophages.

**Figure 6:**
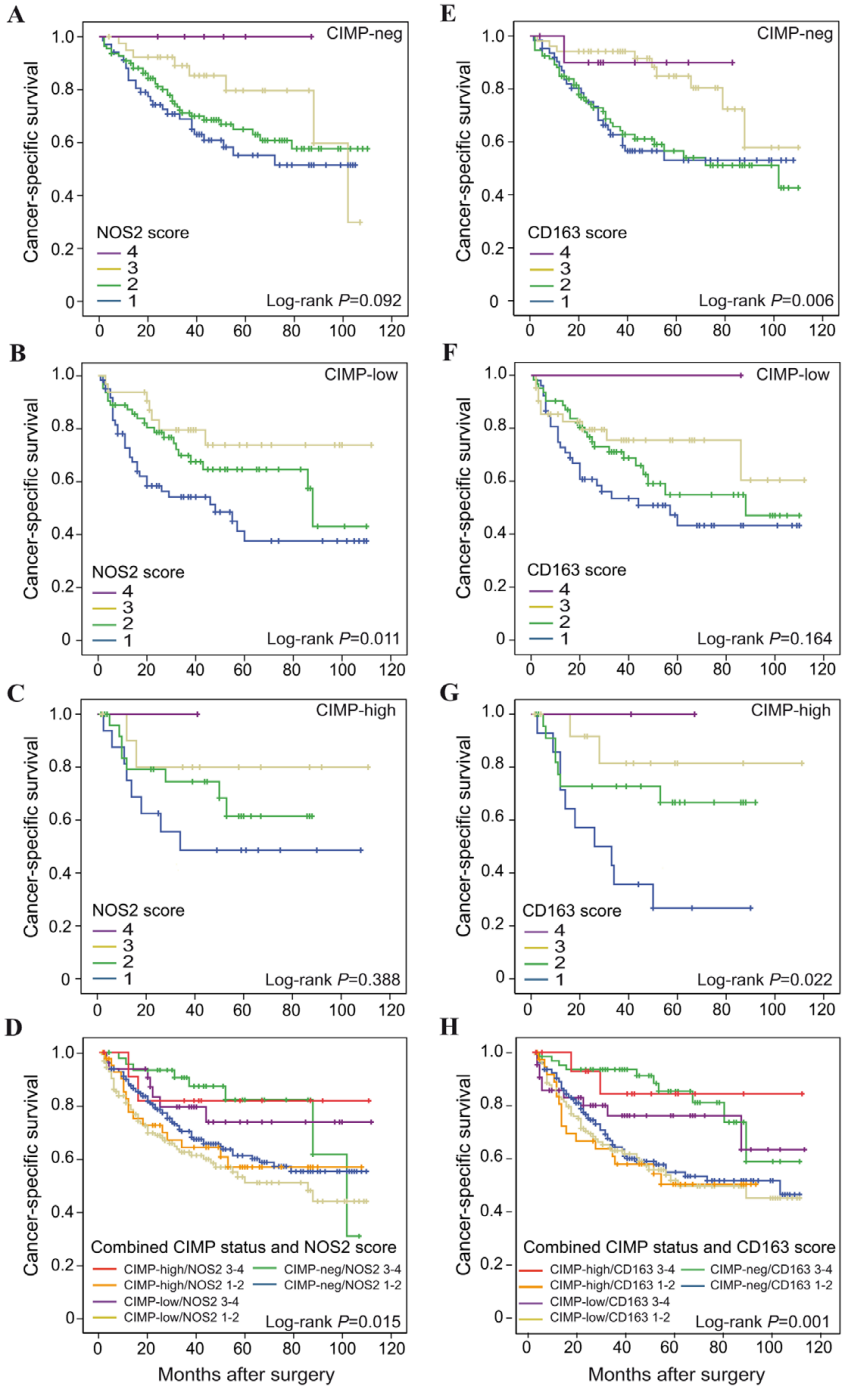
Cancer-specific survival in subgroups of CRC arranged according to CIMP status. Subgroups of CRC were arranged according to CIMP status and scored for NOS2 (A–D) and CD163 (E–H) expression, score 1–4. Shown are Kaplan-Meier plots of specimens of (A and E) CIMP-negative cases, (B and F) CIMP-low cases, (C and G) CIMP-high cases, and (D and H) combined CIMP status and NOS2 or CD163, score 1–2 or 3–4, respectively. Log-rank tests were used to calculate *P* values.

Macrophage infiltration was shown to be of prognostic importance in all CIMP subgroups (Figure 6A–C and E–G). NOS2^+^ macrophage infiltration showed a significant effect on cancer-specific survival in CIMP-low cases (*P* = 0.011). Macrophages expressing CD163 showed significant effects on prognosis in CIMP-neg (Log-rank *P* = 0.006) and CIMP-high (Log-rank *P* = 0.022) cases. When combining CIMP screening status with low (score 1–2) or high (score 3–4) infiltration of macrophages expressing NOS2 or CD163, significant effects on prognosis was found for both NOS2 (Log-rank *P* = 0.015) and CD163 (Log-rank *P* = 0.001) infiltration (Figure 6D and H). The most favourable prognosis was found in highly infiltrated CIMP subgroups (Figure 6D and H). CIMP subgroups with low infiltration of NOS2^+^ or CD163^+^ cells in comparison showed a worse prognosis. No significant differences on prognosis were found between CIMP-negative, CIMP-low or CIMP-high cases within subgroups with low or high infiltration of NOS2^+^ or CD163^+^ macrophages.

## Discussion

The host microenvironment undergoes dramatic changes during the progression of cancer, affecting stromal cells, matrix composition, angiogenesis as well the immune response, which in turn can have significant effects on tumor growth and spread [41]. An inflammatory tumor microenvironment has been suggested as the seventh hallmark of cancer progression [42]. Analysis of the immune contexture - i.e. the location, density and functional orientation of immune cells - and how it is integrated with tumor molecular features can provide important information on patient prognosis as well as prediction of the response to various treatment therapies [43], [44]. Macrophages play an important role at the tumor front, secreting factors that in many ways might affect both the tumor and surrounding stromal cells, including other cells of the immune system.

We have previously shown in a relatively large clinical cohort that a high infiltration of cells expressing the macrophage marker CD68 at the tumor front in CRC results in an improved prognosis [18]. Here, the distribution of macrophages with a M1 or M2 phenotype was evaluated *in situ* in this cohort to analyze for the importance of different subtypes of macrophages in CRC patient prognosis. For this study, NOS2 and CD163 were selected as markers to separate between macrophages displaying primarily M1 or M2 phenotypes, respectively. Both NOS2 and CD163 have been utilized by others as markers to define M1 or M2 macrophage phenotypes in human cancers [10], [45]–[57]. We here confirmed with double immunoflourescent staining and confocal analysis that these markers to a large extent do separate between two different cellular subpopulations that are of the macrophage lineage (Figure 1). However, there appears to be a small number of cells that do express both NOS2 and CD163, but most often in reduced amounts, suggesting that a mixed phenotype sometimes occur. Macrophage subtypes that highly express either NOS2 or CD163, however very rarely express the marker for the opposite subtype. The distinct definition of macrophages in to populations of M1 and M2 subtypes is likely to be a slight oversimplification, since macrophages are highly plastic cells and can display a spectrum of phenotypes [58]. However, markers of M1 and M2 macrophages can still be used to recognize the main phenotype or function of different macrophage populations. Even though we find that NOS2 and CD163 are expressed by different populations of macrophages, there is still a risk that not all M1 or M2 macrophages express these markers and that we therefore might lose parts of the macrophage populations in our study. Further studies are needed to verify the M1 and M2 phenotypes and to find more specific markers that distinguish between M1 and M2 macrophage populations.

When correlated to clinicopathologic characteristics (Table 2), a weak linear trend was found for increased infiltration of M1 macrophages from the ceacum to the rectum, which is in line with the colorectal continuum theory proposed by Yamauchi et al [59], [60]. In their study, CIMP-high, MSI-high and *BRAF* mutations were found to gradually increase from the rectum to the ascending colon. Ceacal cancers were found to represent a unique subtype that did not follow the linearity trend. However, for macrophage infiltration ceacal cancers were not excluded from linearity. Expression of NOS2 and CD163 inversely correlated to tumor stage, indicating that higher stage tumors to a larger extent have escaped the immune system. Furthermore, NOS2 and CD163 expression correlated well to expression of the macrophage marker CD68 (*P*<0.0001), which supports that NOS2 and CD163 are expressed by cells of the macrophage lineage.

In many clinical studies it has been observed that a high infiltration of TAMs correlates to a poor prognosis and TAMs are thus suggested to be of M2 phenotype that promote tumor progression. However, we and others have recently seen that high amounts of TAMs in CRC impart a better prognosis and survival rate [17]–[20], [52]. This is the first study, to our knowledge, where markers for subtypes of M1 and M2 macrophages are used together in purpose to compare the distribution of different macrophage phenotypes and their relation to prognosis in CRC. We could observe a significant statistical correlation between the amount of NOS2^+^ and CD163^+^ macrophages (*P*<0.0001), demonstrating the parallel presence of macrophages with both M1 and M2 phenotypes at the tumor invasive front (Table 1). Furthermore, an increased infiltration of both NOS2^+^ and CD163^+^ macrophages at the tumor front was correlated to a significantly improved prognosis (Figure 3). This correlation was found also in a subgroup of curatively resected colon cancers, but not in a subgroup of curatively resected rectal cancers. A possible explanation for this difference could be that in contrast to most colon cancer patients, many (60%) of the rectal cancer patients received preoperative radiotherapy, which is known to cause a reduced inflammatory reaction [61]. However, we found no difference in macrophage infiltration between tumors in patients that had received preoperative radiotherapy and those that had not (Table 2).

The relation between infiltrating macrophages of M2 phenotype and prognosis in CRC has been previously analyzed in a few studies. According to Nagorsen et al., like in our study, stromal infiltration of CD163^+^ M2 macrophages in CRC was correlated to a significantly improved survival [52]. These authors did not however evaluate the parallel presence of M1 macrophages. Algars et al. found a positive correlation of peritumoral CLEVER-1/Stabilin-1^+^ M2 macrophages and survival in CRCs [17]. They further found that a low M1/M2 ratio resulted in more recurrent disease. However, in their study M1 macrophages were regarded as those macrophages that did not express Clever-1/Stabilin-1. M1 macrophages have been proposed to have tumoricidal activity, and as expected, patients harbouring tumors with high infiltration of NOS2^+^ macrophages were found to have a significantly better prognosis than those with little or no NOS2 infiltration (Figure 3A). Similar to our study, Ohri et al. performed a study on macrophage distribution in non small cell lung cancer (NSCLC) using, among others, NOS2 as a M1 marker, where the presence of NOS2^+^ macrophages in tumor islets, but not tumor stroma, was associated with an improved prognosis [54]. Also Ma et al. recently published a study where M1 macrophages in tumor islets, as well as tumor stroma, of NSCLC were associated with a better prognosis [51]. However, in their study, unlike our results, no effect on prognosis was seen by CD163^+^ M2 macrophage infiltration, suggesting that in NSCLC, M1 and M2 macrophage infiltration is not correlated to the same extent as in CRC, or that the functions of M2 macrophages may differ between the two cancer forms. This in turn suggests that there are differences in macrophage distribution and function in different types of cancers, which needs to be further evaluated. The concomitant presence of both subtypes of macrophages in CRC suggests that the balance between M1 and M2 macrophages could be important for patient outcome. However, we were unable to find any difference on survival in patients with different ratios of NOS2^+^ to CD163^+^ macrophages (Figure 4). Therefore, we speculate that as long as macrophages that display a M1 phenotype are present, their anti-tumorigenic properties might dominate over the tumor promoting effect of macrophages of a M2 phenotype, resulting in a favourable prognosis in our study patients. Possible explanations for the beneficial effect of TAMs on prognosis in CRC compared to the negative effect of TAMS in some other cancers, could be either that the M1 macrophage phenotype is more prominent in CRC or that the M2 phenotypes have less hazardous tumor promoting effects. It is interesting to speculate that in CRC, these differences might be attributed to the intestinal environment, where functional adaptations of macrophages are necessary to maintain local tissue homeostasis [62]. Further studies are required to address the sublocalization of macrophage phenotypes in CRC and how the intestinal tumor microenvironment might support a continuous M1 macrophage reaction. This in turn could lead to the identification of factors that can be used to manipulate the tumor microenvironment in favour or a M1 macrophage response and prevention of tumor progression.

In an attempt to study if the distribution of M1 and M2 macrophage phenotypes might be affected by mutations or epigenetic changes, we looked at the distribution in well characterized subtypes of CRC. The association between an increased macrophage infiltration and an improved prognosis was found to be independent of MSI screening status and CIMP status (Figure 5 and 6, respectively). MSI CRCs are shown to have an improved prognosis compared to MSS CRCs [33], a difference that did not reach statistical significance in this study. MSI tumors are defect in DNA mismatch repair and as a result of accumulating mutations, MSI CRCs are therefore suggested to be more immunogenic [63], [64]. According to this theory, Bauer et al. recently recognized a significant correlation between CD163-positive macrophage infiltration and MSI screening status in a selected cohort of Lynch syndrome-associated CRCs [65]. Here, we find no evidence for that MSI tumors are more efficiently recruiting macrophages of either M1 or M2 phenotype compared to MSS tumors (Table 3). Furthermore, we found that macrophage infiltration was of prognostic impact in both MSI and MSS cases (Figure 5). The reason why there are differences between their study and ours can be explained by the selection for a high level of microsatellite instability (MSI-H) in Lynch syndrome CRCs. However, we did find significant correlations when combining MSI screening status with NOS2^+^ or CD163^+^ macrophage infiltration, suggesting that MSI screening status and macrophage infiltration might be independent prognostic factors (Figure 5).

We found that infiltration of macrophages with a M1 or M2 phenotype is independent of CIMP status (Table 3). When combining MSI screening status and CIMP status, however, a significant correlation was found for CD163 infiltration and CIMP status in MSS CRCs (Table 3). Furthermore, macrophage infiltration was found to have prognostic impact in all CIMP groups (Figure 6). Also here, significant correlations were found when combining CIMP status with NOS2^+^ or CD163^+^ macrophage infiltration (Figure 6).

In conclusion, we show, in line with the general view of M1 and M2 macrophage functions [7], [9],[58], that high infiltration of M1 macrophages is correlated to a better prognosis in CRC in a stage dependent manner. However, in CRC the increased infiltration of M1 macrophages at the tumor front was found to be accompanied by a concomitant increase in M2 macrophages. We therefore suggest that the presence of M1 macrophages is favourable for survival in patients with CRC, despite the parallel presence of M2 macrophages.
